# Rapid Multi-Sensor Feature Fusion Based on Non-Stationary Kernel JADE for the Small-Amplitude Hunting Monitoring of High-Speed Trains

**DOI:** 10.3390/s20123457

**Published:** 2020-06-18

**Authors:** Jing Ning, Mingkuan Fang, Wei Ran, Chunjun Chen, Yanping Li

**Affiliations:** 1 School of Mechanical Engineering, Southwest Jiaotong University, Chengdu 610031, Sichuan, China; 13971658422@163.com (M.F.); ranwei@swjtu.edu.cn (W.R.); cjchen@swjtu.edu.cn (C.C.); signal2020_swj@163.com (Y.L.); 2 Technology and Equipment of Rail Transit Operation and Maintenance Key Laboratory of Sichuan Province, Chengdu 610031, Sichuan, China

**Keywords:** high-speed trains, hunting, non-stationary, feature fusion, multi-sensor fusion

## Abstract

Joint Approximate Diagonalization of Eigen-matrices (JADE) cannot deal with non-stationary data. Therefore, in this paper, a method called Non-stationary Kernel JADE (NKJADE) is proposed, which can extract non-stationary features and fuse multi-sensor features precisely and rapidly. In this method, the non-stationarity of the data is considered and the data from multi-sensor are used to fuse the features efficiently. The method is compared with EEMD-SVD-LTSA and EEMD-JADE using the bearing fault data of CWRU, and the validity of the method is verified. Considering that the vibration signals of high-speed trains are typically non-stationary, it is necessary to utilize a rapid feature fusion method to identify the evolutionary trends of hunting motions quickly before the phenomenon is fully manifested. In this paper, the proposed method is applied to identify the evolutionary trend of hunting motions quickly and accurately. Results verify that the accuracy of this method is much higher than that of the EEMD-JADE and EEMD-SVD-LTSA methods. This method can also be used to fuse multi-sensor features of non-stationary data rapidly.

## 1. Introduction

Hunting motion is a self-excited vibration that is a serious obstacle to the safety of high-speed trains [1]. Monitoring systems are designed to detect hunting only after it has developed to a specific degree. Besides, in most cases, the recognition result is only obtained using a single observation. Therefore, the accuracy and real-time performance of monitoring systems need to be further improved [2]. With the increasing performance requirements of high-speed trains, it is important to establish an accurate and rapid feature extraction method for hunting detection through multi-characterizations before the phenomenon has developed to any significant degree. 

The structure of high-speed trains is very complicated and their working conditions are very poor, resulting in non-stationary vibration signals [3]. In the existing feature extraction research, a variety of data extraction algorithms are utilized. These algorithms can be roughly divided into two categories: those designed for stationary data and those for non-stationary data. Feature extraction methods for stationary data include Singular Value Decomposition (SVD), Linear Discriminant Analysis (LDA) [4,5], Principal Component Analysis (PCA) [6], Locality Preserving Projection (LPP) [7], and so on. However, it is hard to extract features of non-stationary data using these methods. For non-stationary data, manifold learning is a good feature extraction method [8,9,10,11]; however, it incurs a high computational cost and has extremely long calculation times, which means that the diagnostic information cannot be fed back to the system in time. Therefore, a rapid yet precise method to extract the non-stationary signal in practical engineering applications is needed. Cardoso [12,13] proposed a method named Joint Approximate Diagonalization of Eigen-matrices (JADE) in the field of blind source separation, which is used to quickly separate multiple features. Because the method is simple and effective, it is also widely used in pattern recognition. Liu [14] improved the JADE method and achieved a good feature fusion performance by simplifying the calculations, and then developed a method based on kernel JADE to identify the rolling bearing fault [15]. The method based on JADE was adopted to diagnose the faulty parts of the rolling bearing in [16], and a similar method was applied to predict coaxial bearing performance degradation in [17]. However, in this application, the non-stationary and rapid character of the signal is not considered. 

The structure of high-speed trains is complex. The vibration signals from high-speed trains are affected by many factors, such as the propagation path and the measuring point location, which lead the signal to couple with different characteristic information. Pattern recognition based on single sensor vibration signals has limitations, which cannot completely render the evolutionary characteristics of high-speed train vibrations [18]. Therefore, it is necessary to utilize a multi-sensor fusion method to extract the features of the train’s operating state.

Furthermore, the evaluation parameters of lateral stability of railway passenger trains in different countries are different. Lateral force on the rail and on the wheel axis, lateral acceleration of the bogie frame, acceleration of the axle-box, and lateral acceleration of the vehicle body can be used as evaluation parameters, respectively [19,20,21,22]. However, only one parameter is used in each standard, and this parameter has different limits. For example, the influence of frequency is not considered when lateral acceleration of the bogie frame is used. In fact, the acceleration of the axle-box is a particularly important index in hunting monitoring. Therefore, in this paper, we try to fuse the signal from the bogie frame and axle box to extract the hunting motion features. To monitor the state of hunting of high-speed trains, four basic states are classified: normal, small convergence, small divergence, and hunting. Our aim is to identify the small divergence state before hunting occurs. Thus, in this paper, the monitoring of small amplitude hunting enables us to rapidly distinguish between these four basic states online [23].

Moreover, for real-time classification, it is extremely important to ensure that once the small amplitude appears to diverge, the entire calculation process can be completed immediately. Therefore, the time of the whole calculation process must be short enough for real-time classification.

Considering the above problems, a rapid multi-sensor feature fusion method based on the Non-stationary Kernel JADE method is proposed. The JADE method is a fast and accurate feature fusion algorithm, but it is generally used in stable environments. In order to use the algorithm in a non-stationary environment, the whole time series is divided into *M* time periods [24] and the kernel function is introduced. Then, *M* kernel matrices are obtained by the *M* time periods, which are jointly decomposed. After this, Jacobian rotation is used to obtain the unitary matrix by diagonalizing multiple kernel matrices simultaneously to extract the non-stationary fusion features. In addition, in order to visualize the data features, the extracted fusion features are expressed in three dimensions. The between-class indicator and within-class indicators [25] are also employed to describe the clustering performance of the features quantitatively.

In this paper, a multi-sensor data feature extraction framework is provided, in which a rapid feature fusion method using Non-stationary Kernel JADE (NKJADE) is proposed. This framework consists of the following series of steps. First, the Ensemble Empirical Mode Decomposition (EEMD) method is utilized to decompose the preprocessed signals to Intrinsic Mode Functions (IMFs). Then, the energy matrices are obtained using the IMFs and the fusion features are obtained through NKJADE, followed by inputting the extracted features to an LSSVM [26,27] for training and recognition.

Data from Case Western Reserve University (CWRU) are used to verify the performance of this method against that of the SVD and JADE methods. In this paper, the proposed method is also applied to identify the evolutionary trend of the hunting motion quickly and accurately. The results verify that the accuracy of this method is much higher than that of the JADE method. 

The remainder of this paper is organized as follows: In Section 2, the theoretical backgrounds of Ensemble Empirical Mode Decomposition (EEMD) [28] and a separability metric are introduced. The proposed method of non-stationary Kernel JADE is also described. The framework of the proposed method is introduced in Section 3. In Section 4, data from Case Western Reserve University (CWRU) are used to test the proposed method, and the operational data of multiple states of high-speed trains are used to verify the accuracy and rapidity of the proposed method. Finally, the conclusion is presented in Section 5.

## 2. Theoretical Background

### 2.1. EEMD

The basic idea of the EEMD method is to use the sifting process to decompose the signal into several intrinsic mode functions, when Gaussian white noise is added. For the original signal, the specific steps to use EEMD to decompose it into IMFs are as follows:

(1) Obtain the overall signal by adding Gaussian white noise to the original signal:
  (1) $$a^{\prime }(t)=a(t)+\omega (t)$$
(2) The overall signal is decomposed to obtain the IMF components of each order, where *i* represents the *i*-th component, *r* is the residual term, and *n* is the number of IMFs:
  (2) $$a^{\prime }(t)=\sum_{i}^{n}c_{i}+r$$
(3) Repeat step (1) and (2), each time adding different white noise sequences of the same amplitude:
  (3) $$a_{j}^{\prime }(t)=\sum_{i=1}^{n}c_{ij}+r_{j}$$
  In Equation (3), $c_{ij}$ is the *i*-th IMF component of the decomposition to which white noise at for the *j*-th time, while $r_{j}$ is the residual value of the decomposition.
(4) Using the zero-mean principle of the Gaussian white noise frequency, the effect of white noise can be eliminated, and the IMF component corresponding to the original signal can be expressed as:
  (4) $$c_{i}(t)=\frac{1}{N}\sum_{j=1}^{N}c_{ij}(t)$$
  where *n* represents the number of times the white noise is added and $c_{i}$ represents the *i*-th IMF component obtained by the EEMD decomposition of the original signal.

### 2.2. IMF Energy Matrix

For the IMF component $c_{i}^{\prime }=[c_{i}^{\prime }(1),\ldots \ldots ,c_{i}^{\prime }(M)]$, the energy feature can be expressed as:

(5) $$e_{i}=\sum_{j=1}^{M}{\left[c_{i}^{\prime }(j)\right]}^{2}$$

All IMF components of a vibration signal form a feature energy vector $e=[e_{1},e_{2},\ldots \ldots e_{n}]$, where *n* is the dimension of vector e and represents the number of IMF components. 

### 2.3. Proposed Non-Stationary Kernel JADE

Since the original JADE algorithm is based on stationary signal analysis, considering the non-stationary nature of high-speed train signal, the kernel JADE method [15] is applied to a non-stationary environment.

The main idea of the kernel is to map the input matrix into the nonlinear space Φ Suppose $X=\{x_{1},x_{2},\ldots ,x_{m}\}$; then, the processing can be defined as follows:

(6) $$\left\{x_{1},x_{2},\ldots ,x_{m}\}\to \{\Phi (x_{1}),\Phi (x_{2}),\ldots ,\Phi (x_{m})\right\}$$

During implementation, we need to calculate the inner product of two eigenvectors which have been mapped into a nonlinear space using a kernel function, and a kernel matrix will be calculated using Equation (7):

(7) $$K_{ij}=k(x_{i},x_{j})=<\Phi (x_{i}),\Phi (x_{j})>$$

where $x_{i},x_{j}$ are vectors. The commonly used kernel function includes the following [29]:

(8) $$k(x_{i},x_{j})=\exp(-\frac{{\left\|x_{i}-x_{j}\right\|}^{2}}{2\delta^{2}})$$

(9) $$k(x_{i},x_{j})={\left(\alpha x_{i}{}^{T}x_{j}+c\right)}^{d}$$

(10) $$k(x_{i},x_{j})=\tanh(\alpha x_{i}{}^{T}x_{j}+c)$$

Similar to KPCA [30], the centered kernel matrix ***K*** can be calculated through Equation (11):

(11) $$K_{ij}=K_{ij}-\frac{1}{M}\sum_{r=1}^{M}K_{ir}-\frac{1}{M}\sum_{r=1}^{M}K_{rj}+\frac{1}{M^{2}}\sum_{r,s=1}^{M}K_{rs}$$

The whole time series is divided into *M* segment intervals $T_{1},\ldots \ldots ,T_{M}$, and consequently *M* covariance matrices $S_{T_{1}},\ldots \ldots ,S_{T_{M}}$ can be generated. These *M* covariance matrices are jointly diagonalized to find a unitary matrix ***U*** which can diagonalize *M* covariance matrices simultaneously; then, the energy features can be extracted.

**Step 1:** For *M* segment intervals $T_{1},\ldots \ldots ,T_{M}$, the covariance matrices of signal *x*(*t*) can be expressed as:

(12) $$Cov_{T_{k}}=S_{T_{k}}=\frac{1}{\left|T_{m}\right|}\sum_{t\in T_{m}}\left[k(s_{t},s_{t})\right]$$

where $s_{t}=x_{t}-E(x_{t})$, $E(x_{t})$ denotes the mean of $x_{t}$.

**Step 2:** The most common method to diagonalize matrices $S_{T_{1}},\ldots \ldots ,S_{T_{M}}$ is to diagonalize the first matrix, and then transform the remaining *M*−1 matrices into diagonalization. **W** is the diagonalized matrix of the covariance matrix $S_{T_{1}}$:

(13) $$W=S_{T_{1}}^{-1/2}=V^{H}\Lambda^{-1/2}V$$

In Equation (13), **V** is the eigen-matrix of $S_{T_{1}}$, while Λ is the eigenvector of $S_{T_{1}}$.

For the remaining *M*−1 matrices, the diagonalization matrix can be defined respectively:

(14) $$S_{T_{m}}^{*}=S_{T_{1}}^{-1/2}S_{T_{m}}{\left(S_{T_{1}}^{-1/2}\right)}^{H},m=2,\ldots ,M$$

**Step 3:** The approximate joint diagonalization problem is equivalent to finding an orthogonal matrix **U** that minimizes:

(15) $${\sum_{m=2}^{M}\left\|\mathrm{off}(US_{T_{m}}^{*}U^{H})\right\|}^{2}=\sum_{m=2}^{M}\sum_{b\neq d}{\left(US_{T_{m}}^{*}U^{H}\right)}_{bd}^{2}$$

where $\mathrm{off}(US_{T_{m}}^{*}U^{H})$ has the same off-diagonal elements as $US_{T_{m}}^{*}U^{H}$, and the diagonal element is zero, while *b* and *d* represent the *b*-th row and *d*-th column of the matrix, respectively. 

Since the sum of squares remains the same when multiplied by an orthogonal matrix, the problem is equivalent to maximizing the sum of squares of the diagonal elements:

(16) $${\sum_{m=2}^{M}\left\|\mathrm{diag}(US_{T_{m}}^{*}U^{H})\right\|}^{2}=\sum_{m=2}^{M}\sum_{b=1}^{p}(US_{T_{m}}^{*}U^{H}{)}_{bb}^{2}$$

where *p* represents the dimension to which the feature is extracted.

**Step 4:** Givens rotation is used to transform the set of matrices to a more diagonal form, two rows and two columns at a time. The Givens rotation matrix is given by:

(17) $$G(i,j,\theta )=\left(\begin{array}{ccccccc} 1 & \cdots & 0 & \cdots & 0 & \cdots & 0\\ \vdots & \ddots & \vdots & & \vdots & & \vdots \\ 0 & \cdots & \cos(\theta ) & \cdots & -\sin(\theta ) & \cdots & 0\\ \vdots & & \vdots & \ddots & \vdots & & \vdots \\ 0 & \cdots & \sin(\theta ) & \cdots & \cos(\theta ) & \ldots & 0\\ \vdots & & \vdots & & \vdots & \ddots & \vdots \\ 0 & \cdots & 0 & \cdots & 0 & \cdots & 1 \end{array}\right)$$

where

(18) $$\theta =\frac{1}{2}\mathrm{arccot}\left[\frac{{\left(S_{T_{m}}^{*}\right)}_{22}-{\left(S_{T_{m}}^{*}\right)}_{11}}{2{\left(S_{T_{m}}^{*}\right)}_{12}}\right]$$

The initial value for the orthogonal matrix *U* is the identity matrix *I*. Matrices $S_{T_{2}},\ldots ,S_{T_{m}}$ are then updated using:

(19) $$S_{T_{m}}^{*}\leftarrow G(1,2,\theta )S_{T_{m}}^{*}G(1,2,\theta ),m=2,\ldots ,M\;U\leftarrow UG(1,2,\theta )$$

When the values of all non-diagonal elements are less than a given threshold ε, the iteration is completed, and the joint approximation diagonalization is achieved. The unitary matrix $\hat{U}$ is obtained so that multiple matrices are diagonalized jointly.

**Step 5**: The transform matrix *A* can be calculated as ${A=\hat{U}W}^{\#}$, where superscript # denotes the pseudo-inverse. 

As such, the original signal *s*(*t*) can be expressed as:

(20) s(t)=A⋅K

Since the NKJADE method can extract the nonlinear relationships hidden in the high-dimensional feature space, the fusion features can be estimated through the joint feature decomposition using multiple inputs. The fusion features can express the nonlinear and non-stationary relationships hidden in the inputs well, so they can represent the characteristic relationship of different states, and then distinguish different feature states quickly and accurately. 

### 2.4. Separability Evaluation 

To illustrate the merit of our proposed algorithm, the separability *J* is utilized to demonstrate the algorithm’s ability to form distinct classes. The capability of the feature extraction in pattern classification can be described quantitatively using between-class scatter $S_{b}$, within-class scatter $S_{w}$ [31], and separability [32]. Assuming that the data have a total of C classifications, the vector of the *i*-th classification is:

(21) $$x^{i}=\left(x_{1}^{i},x_{2}^{i},\ldots ,x_{n_{i}}^{i}\right)$$

where $n_{i}$ is the number of *i*-th classification.

Between-class scatter $S_{b}$ and within-class scatter $S_{w}$ can be calculated as follows:

(22) $$S_{b}=\sum_{i=1}^{C}p_{i}(m_{i}-m){\left(m_{i}-m\right)}^{T}$$

(23) $$S_{w}=\sum_{i=1}^{C}\left[\frac{p_{i}}{n_{i}}\sum_{k=1}^{n_{i}}(x_{k}^{i}-m_{i}){\left(x_{k}^{i}-m_{i}\right)}^{T}\right]$$

where $p_{i}=n_{i}/\sum_{j=1}^{C}n_{j}$, $m_{i}=mean(x_{n_{i}}^{i})$, $m=\sum_{i=1}^{C}p_{i}m_{i}$.

The between-class scatter $S_{b}$ describes how far different classes are separated, and the within-class scatter $S_{w}$ indicates how compactly each class of samples is distributed. Based on between-class scatter and within-class scatter, separable evaluation *J* is introduced to describe the clustering ability of different methods. Separable evaluation *J* could be calculated as follows:

(24) $$J=\mathrm{trace}(S_{b}/S_{w})$$

where function *trace* refers to the sum of diagonal elements. 

## 3. Methodology

**Figure 1:**
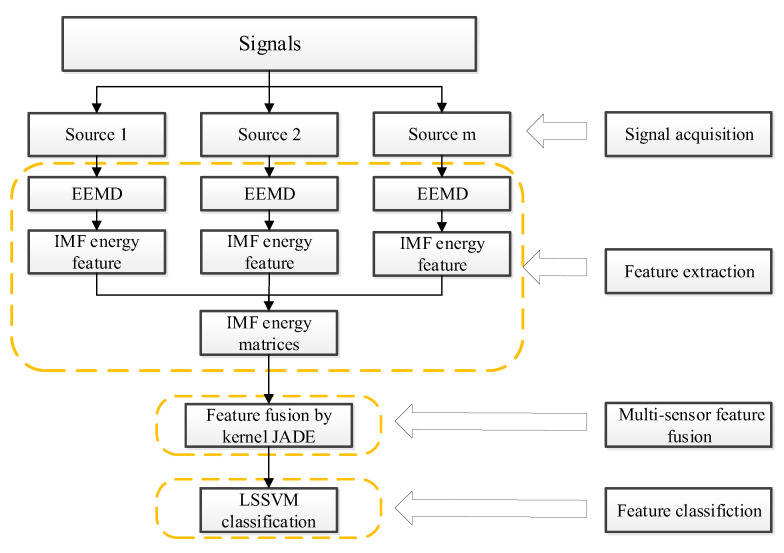
Method framework.

For the different characteristics of vibration signals, a multi-sensor data feature extraction framework is provided, in which a rapid feature fusion method using NKJADE is proposed. The framework of the feature extraction method is shown in Figure 1. The method can quickly and accurately extract different features of information contained in non-stationary vibration signals. The main steps of this framework are as follows:

(1) The *m* position sensor data and *l* classes are selected, and a total of *m* sample matrices are obtained.
(2) The EEMD algorithm is used to separately decompose the sample matrices of *m* positions, then *n* IMF components of each sample signal can be obtained.
(3) By extracting the energy features of the *n* components of IMF obtained using decomposition, an IMF energy matrix is obtained from each position. In this paper, a total of *m* energy matrices of IMF were obtained.
(4) For the obtained energy matrices of IMF of *m* positions, a rapid feature fusion method using NKJADE is proposed, so the dimensionality is reduced to three for a better-observed performance.
(5) The LSSVM is trained on and used to test the fusion features to verify the accuracy of the method.

## 4. Experiment Results and Analysis

In order to verify the valid of the method, we applied it to bearing fault identification (Case I) and small amplitude hunting monitoring of high-speed trains (Case II). Some traditional approaches, such as Ensemble Empirical Mode Decomposition Joint Approximate Diagonalization of Eigen-matrices (EEMD-JADE) and Ensemble Empirical Mode Decomposition Singular Valuable Decomposition Learning Technology Systems Architecture (EEMD-SVD-LTSA), were compared with the proposed method. 

### 4.1. Case I—Case Western Reserve University Data

#### 4.1.1. Data Description

The bearing test data for normal and faulty bearings were from the Case Western Reserve University (CWRU). The test signal contained normal state, ball fault, and inner and outer race faults; for the latter three, the fault diameters were 0.07, 0.14 and 0.21 inches, respectively.

The data used in this paper were collected from the drive end. The sampling frequency was 12 KHZ and the speed of the shaft was 1725 r/min, corresponding to 400 points collected per revolution. In order to reduce the influence of equipment fluctuations, each sample contained 800 points.

As shown in Table 1, two datasets were selected. Dataset A had the same fault location (inner race fault), but the fault size was different. Dataset B had different fault locations, but the fault size was the same, and the outer race fault was at the location of 3 o’clock. Each of the datasets was divided into three categories.

#### 4.1.2. Signal Processing Results

**Figure 2:**
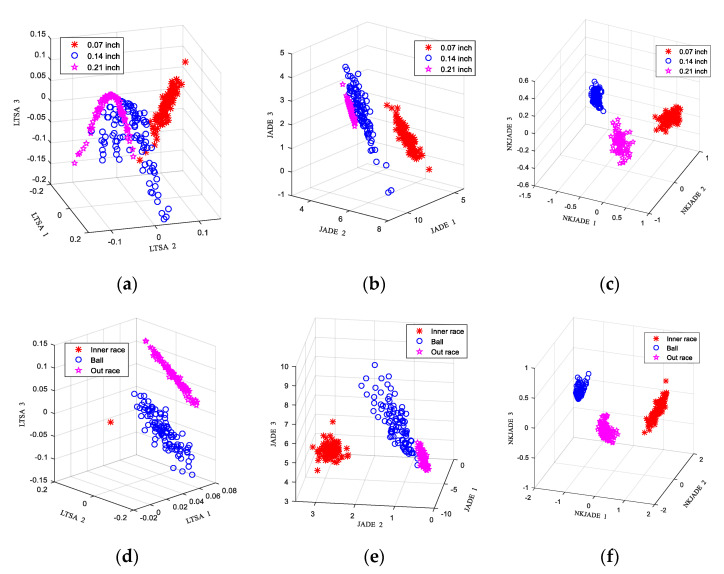
Scatter plots of faults. (**a**) EEMD-SVD-LTSA on dataset A; (**b**) EEMD-JADE on dataset A; (**c**) EEMD-NKJADE on dataset A; (**d**) EEMD-SVD-LTSA on dataset B; (**e**) EEMD-JADE on dataset B; (**f**) EEMD-NKJADE on dataset B.

**Figure 3:**
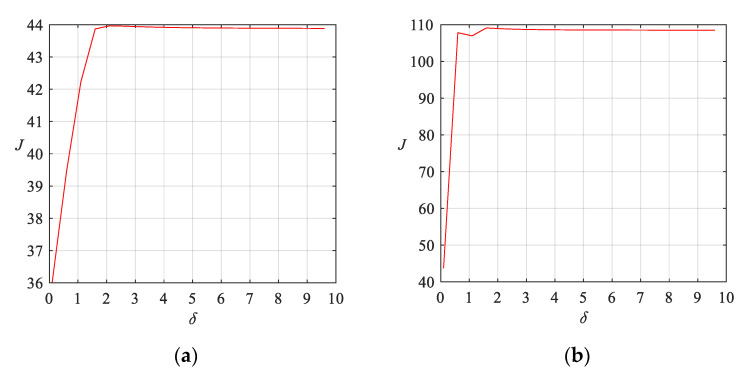
The results of selecting parameters for the Gaussian kernel. (**a**) Parameter selection results of Gaussian kernel of EEMD-NKJADE method on dataset A; (**b**) Parameter selection results of Gaussian kernel of EEMD-NKJADE method on dataset B.

The scatter plots obtained by applying the EEMD-SVD-LTSA, EEMD-JADE method and EEMD-NKJADE methods on dataset A are shown in Figure 2a–c, while the corresponding scatter plots for dataset B are shown in Figure 2d–f. The results of selecting parameters for kernel are shown in Figure 3. The results of the application of the three algorithms in dataset A are shown in Table 2, while the results for dataset B are shown in Table 3.

#### 4.1.3. Discussion

We found that the generalization ability of the Gaussian kernel is better than that of the other kernels. Therefore, we focused on the parameters of the Gaussian kernel.

According to the results of selecting parameters in [15], we tried to set the range of *σ* to (1, 10). The parameter was incremented step by step (parameter step is 0.5, shown in X-axis), and the separability *J* was calculated (shown in Y-axis). The larger *J* is, the better the classification result will be. Therefore, for the parameter selection of EEMD method, the optimal parameter of the Gaussian kernel on dataset A and dataset B is 2.

From Figure 2 and Table 2, we see that all three methods could extract the features with satisfactory results. The accuracy of the three methods was nearly 100% in all cases. The result may be attributed to the CWRU bearing fault data having great differences, which are quite easy to classify. 

However, the separability *J* in Table 2 is different, which is consistent with (a)–(c) in Figure 2. In this respect, the classification ability of EEMD- NKJADE algorithm is obviously better than other algorithms. In addition, the results of the running time in Table 2 show that the time required for EEMD-JADE calculation was relatively short (the PC configuration was as follows: Intel Core i5-4460, 12GB of memory, NVIDIA GeForce GT720).

In order to further verify the robustness of the algorithm, we also tested the results of the algorithm on dataset B.

In Table 3, the accuracy of the three methods in dataset B was almost the same as that in dataset A. However, the separability of dataset B was the highest after being processed using the EEMD-SVD-LTSA algorithm, while in dataset A, the separability achieved using EEMD-SVD-LTSA was the lowest, which indicates that the selection of dataset had a greater impact on EEMD-SVD-LTSA. Compared with EEMD-SVD-LTSA, the results from the EEMD-NKJADE and EEMD-JADE algorithms were less affected by the different dataset, and therefore seem to be more robust. Therefore, the method of EEMD-NKJADE offers superior performance with respect to the classification effect and the robustness, but its calculation time is longer than EEMD-JADE.

### 4.2. Case II—Small Amplitude Hunting Monitoring of High-Speed Trains

#### 4.2.1. Problem Description

**Figure 4:**
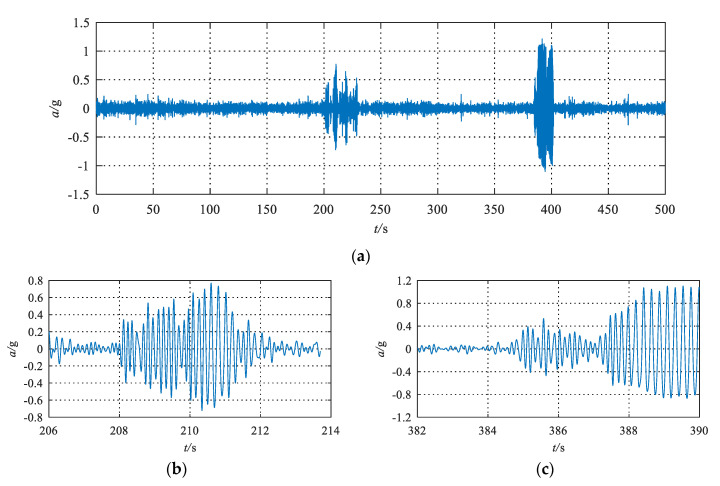
Lateral acceleration signal of the bogie frame when small-amplitude hunting motion occurs during an online test. (**a**) Bogie frame acceleration; (**b**) Small amplitude convergent hunting; (**c**) Small amplitude divergent hunting.

The stability of hunting has always been a key problem in the study of vehicle lateral motion stability [33]. Small amplitude hunting is a sign of hunting instability. In China, hunting phenomena are considered to occur when the amplitude of lateral acceleration signals from the bogie frame reaches or exceeds 8–10 m/s^2^ more than 5 times continuously after a 10 Hz low-pass filter [34]. In Figure 4, the lateral acceleration of the bogie frame signals will sometimes go through a normal operation/small amplitude hunting/normal operation periodic cycle, which is a gradually convergent process. The hunting amplitude in this situation is small and convergent. Sometimes, the signals will go through a normal operation/small amplitude hunting/critical hunting process, which is a gradually divergent process. The hunting amplitude in this situation starts small and then diverges.

Therefore, it is necessary to extract the features of different states rapidly and accurately, especially the small amplitude divergent hunting states, to guarantee the system is alerted in time to ensure the safe operation of the train.

#### 4.2.2. Data Acquisition 

The data used in this paper were lateral acceleration signals of the bogie frame and axle box from an online tracking experiment. The CRTS II ballastless track and seamless rail were used on the whole line. The speed of the train was 320–350 km/h. The sampling frequency was 2500 Hz. All the data were acquired in accordance with China’s Railway Passenger Traffic Safety Monitoring Standard [35]. Although in China the amplitude of lateral acceleration signals from the bogie frame is used as the testing parameter to monitor hunting motion, research has proven that many other testing parameters are also important for hunting monitoring. As such, in this paper, acceleration signals of the bogie frame and the axle box were used. 

**Figure 5:**
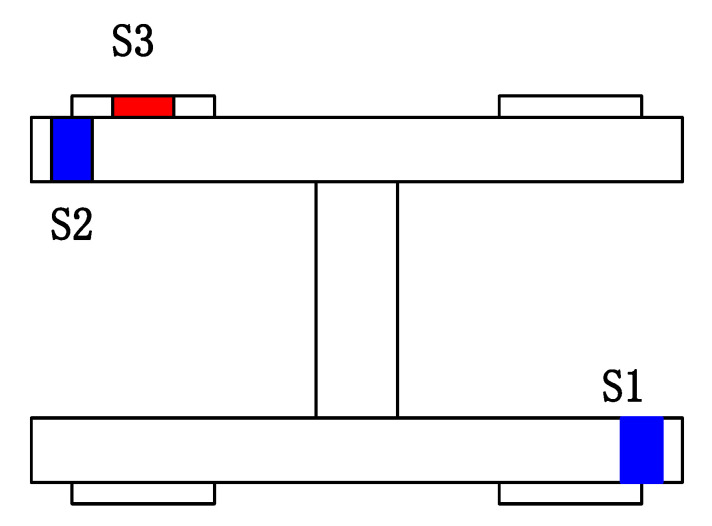
Installation locations of the accelerometers. S1, S2: Accelerometer on the bogie frame; S3: Accelerometer on the axle box.

**Figure 6:**
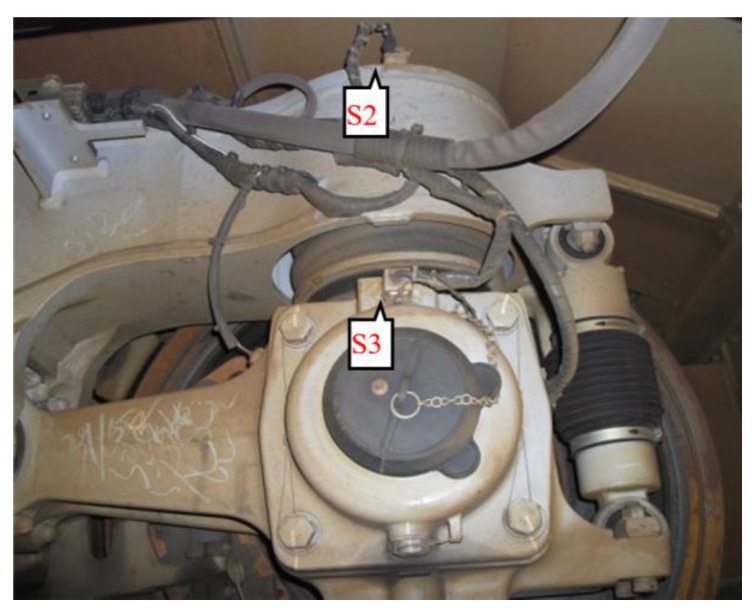
Site installation drawing.

The installation locations of the accelerometers are shown in Figure 5, where two accelerometers are installed in the diagonal direction on the H-shaped bogie frame. The lateral accelerometers from the bogie are respectively denoted as S1 and S2. Also, considering that the vibration of the axle box is important for hunting [36], a sensor located on the axle box was used. In Figure 6, the lateral accelerometer on the axle box is denoted as S3. Figure 5 shows a photograph of the installation site.

Considering the applications of other researchers, the sampling frequency of the original signal was set to 2500 Hz. However, for hunting, the characteristic frequency of the lateral acceleration of the frame is only 3–7 Hz. Therefore, in the preprocessing stage, a 250 Hz resampling method was applied [37], and a low-pass filter of 0–10 Hz was applied. Then, a zero-mean smoothing process was used for preprocessing to eliminate trend terms. In accordance with commonly followed practices, parameters such as the amplitude of the lateral acceleration signals from the bogie frame and axel box were used to obtain a synthetic assessment of the lateral stability of the high-speed train tested. In this paper, the filtered lateral acceleration signals were divided into four states: normal, small amplitude convergent hunting, small amplitude divergent hunting, and hunting. Ten groups of sample data were used for each of the four states and each sensor, yielding a total of 120 sample groups. The length of each sample was 500 points, corresponding to a sample time of 2 s. 

Nonlinear factors [38,39] have been proven to affect the bifurcation evolution of small amplitude hunting and, according to [23], all the values of the Lyapunov exponent of the lateral acceleration are greater than 1, which means that the lateral acceleration signals from the bogie frame have non-stationary characteristics.

#### 4.2.3. Feature Fusion

**Figure 7:**
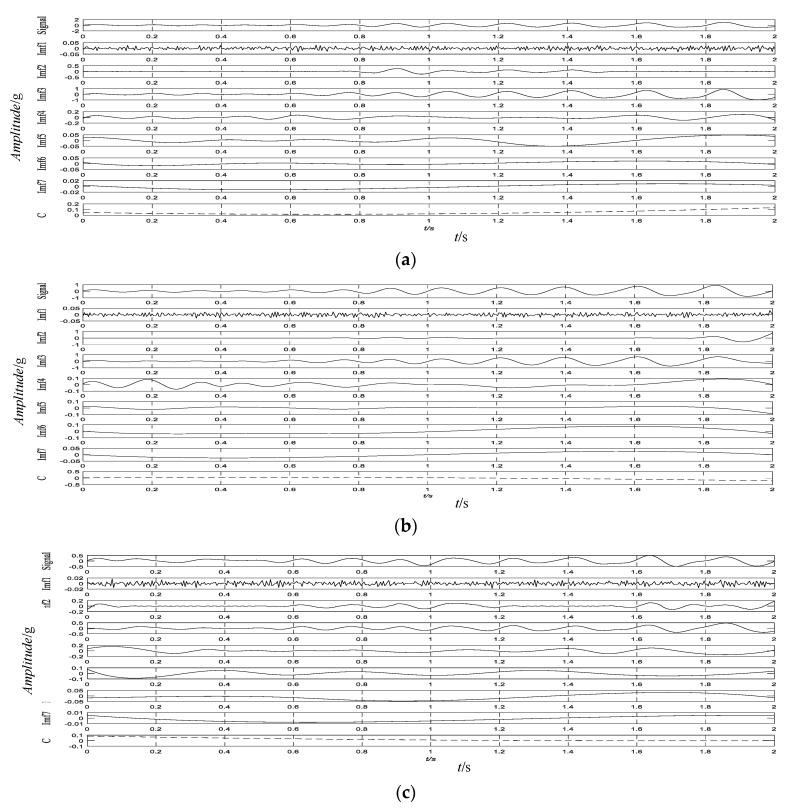
Ensemble Empirical Mode Decomposition (EEMD) diagram of signals at different positions. (**a**) S1 position (bogie frame); (**b**) S2 position (bogie frame); (**c**) S3 position (axle box).

First, EEMD was applied on each signal. Then, 7 IMFs and 1 residue were obtained using the EEMD method. Figure 7 shows an EEMD decomposition view of the three signals at different points during the same time period.

The samples of the three sensors were processed, and the three corresponding energy matrices E1, E2, and E3, with a size of 40 × 8 were obtained. The NKJADE method was used to fuse the three high-dimensional energy matrices and the data dimensionality was reduced. 

### 4.3. Result and Discussion 

#### 4.3.1. Single Sensor Classification Using NKJADE

**Figure 8:**
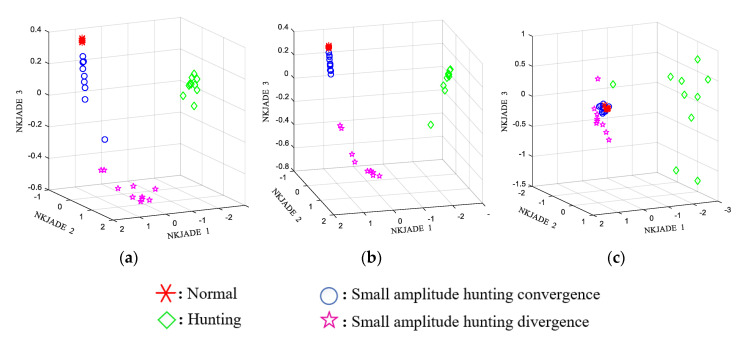
Scatter plot of feature extraction from single sensor using Non-stationary Kernel JADE (NKJADE). (**a**) S1 (bogie frame); (**b**) S2 (bogie frame); (**c**) S3 (axle box).

A scatter plot of the features extracted using a single sensor and NKJADE is shown in Figure 8. The separability *J* and accuracy of the NKJADE method are shown in Table 4.

From Table 4, the accuracy achieved using S1 data only was 97.23%, which was greater than that attained at the other sensor locations. However, when the three sensors were used together, the accuracy rate became 100%, and *J* reached a much greater value. 

#### 4.3.2. Multi-Sensor Fusion Using NKJADE

**Figure 9:**
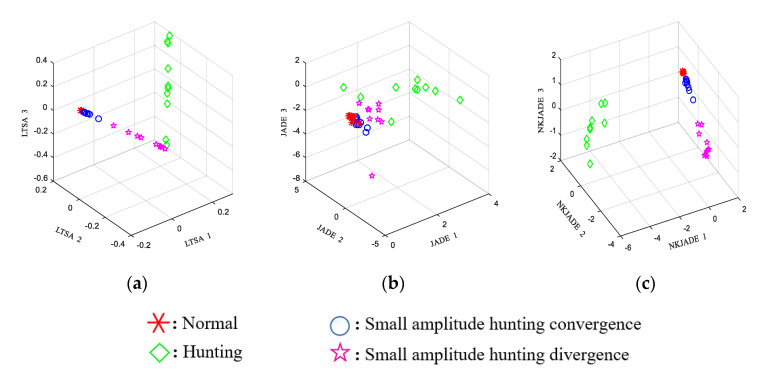
Scatter plot of feature extraction from multi-sensor data. (**a**) EEMD-SVD-LTSA; (**b**) EEMD-JADE; (**c**) EEMD-NKJADE.

The EEMD-SVD-LTSA and EEMD-JADE methods were used to compare the identification accuracy and calculation speed of the feature fusion method in multi-sensor conditions. The results of the methods are shown in Figure 9. The separability *J* and accuracy obtained using the different sensor fusion methods with data from all three sensors are shown in Table 5.

The JADE and SVD-LTSA methods were compared with the proposed method in the multi-sensor feature fusion conditions. From Table 5, the accuracy rate of the SVD-LTSA method was 93.75%, which used the non-stationary method LTSA. The accuracy rate of the JADE method was only 30.12%, which used the stationary method. The accuracy rate of NKJADE (Gaussian kernel, *δ* = 0.6) was 100%, while the separability *J* of NKJADE was greater than those of the other methods. Considering that data from high-speed trains have more typical no-stationary characteristics than the bearing fault data from CWRU, this result shows that the proposed method may more be suitable for non-stationary data. The separability *J* of the JADE method was only 26.67, which is even lower than that achieved using single sensor S2 or S3 data. The reason for this is probably that in the JADE method, the non-stationary condition is not considered. 

Besides, because real-time processing is a significant factor for the small amplitude hunting monitoring of high-speed trains, the calculation time is a very important factor. If the calculation time is too long, the diagnostic information cannot be fed back to the system in time. Compared with the SVD-LTSA method, fusion features can be extracted very quickly using the proposed NKJADE method. The run time of the NKJADE method was nearly the same as that of the JADE method, and the separability of *J* of NKJADE was greater than JADE. This shows that NKJADE is a rapid multi-sensor feature fusion method based on non-stationary condition, which outperforms the SVD-LTSA and JADE methods. It can be applied to the small amplitude hunting bifurcation evolution monitoring in high-speed trains.

**Figure 10:**
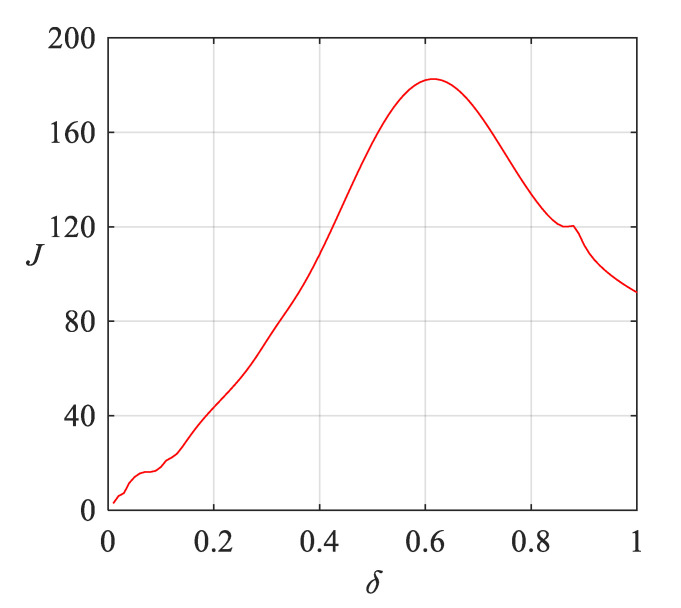
Parameter selection results of Gaussian kernel of EEMD-NKJADE method.

As shown in Figure 10, the parameter is incremented step by step (parameter of step is 0.01, shown in X-axis), and the separability index *J* is calculated (shown in Y-axis). The larger *J* is, the better the classification result will be.

Because the raw data collected are mainly distributed in the range of −1–1 g (gravity). In Equation (8), *σ* is the width parameter of the kernel function, so we tried to set the range of *σ* to (0, 1). As shown in Figure 10, with the increase of *δ, J* increases at first and then decreases. It indicates that the range of parameter selection is reasonable. When *δ* = 0.6, the classification result is the best. 

## 5. Conclusions 

In this paper, a rapid multi-sensor feature fusion method based on NKJADE is proposed, with which the features of multiple sensors can be extracted quickly and accurately. In order to use the algorithm in a non-stationary environment, the whole time series is divided into *M* time periods, and the kernel function is introduced. Then, Jacobian rotation is used to obtain the unitary matrix by diagonalizing multiple kernel matrices simultaneously to extract the non-stationary fusion features.

Our main findings are that:

1. The fusion features can be extracted quickly and efficiently using the proposed method NKJADE compared to the SVD-LTSA and the JADEs methods with bearing fault data from Case Western Reserve University.
2. The NKJADE method can extract the fusion features from non-stationary data effectively compared to the JADE method. In case I, the accuracy rate of the three methods (SVD-LTSA, JADE, and NKJADE) was nearly the same (100%), but in case II, the accuracy rate of the three methods was very different.
3. The NKJADE method can extract the multi-sensor fusion features effectively. The data from hunting monitoring of high-speed trains were used to verify the validity of the method in multi-sensor conditions.

## Figures and Tables

**Table 1:** Description of dataset.

Datasets	Class	Number of Training Samples	Number of Testing Samples	Label
Dataset A	Inner race, 0.07”	70	30	1
	Inner race, 0.14”	70	30	2
	Inner race, 0.21”	70	30	3
Dataset B	Inner race, 0.07”	70	30	1
	Ball, 0.07”	70	30	4
	Outer Race, 0.07”	70	30	5

**Table 2:** Results from dataset A.

Method	EEMD-SVD-LTSA	EEMD-JADE	EEMD-NKJADE
Accuracy (%)	98.33	98.89	100
*J*	36.26	39.24	43.91
Time (s)	1.7593	0.7820	1.0070

**Table 3:** Results from dataset B.

Method	EEMD-SVD-LTSA	EEMD-JADE	EEMD-NKJADE
Accuracy (%)	100	98.89	100
*J*	1.195 × 10^24^	47.96	87.96
Time (s)	1.5513	0.7190	0.9650

**Table 4:** Separability *J* running time and accuracy of NKJADE method.

Sensors	Only S1	Only S2	Only S3	S1, S2 and S3
*J*	65.6	61.1	21.1	155.7
Accuracy (%)	97.23	96.54	29.85	100
Run Time (s)	0.0392	0.0415	0.0387	0.1293

**Table 5:** The separability *J*, running time, and accuracy using different sensor fusion methods.

Method	EEMD-SVD-LTSA	EEMD-JADE	EEMD-NKJADE
*J*	52	26.67	155.7
Accuracy (%)	93.75	30.12	100
Run time (s)	0.9972	0.1216	0.1298
